# Supplementary material for: COVID-19 vaccination and leprosy–A UK hospital-based retrospective cohort study
Source: PLoS Negl Trop Dis. 2023 Aug 4;17(8):e0011493. doi: 10.1371/journal.pntd.0011493 (PMC10431605; doi:10.1371/journal.pntd.0011493)
Supplement: Attachment — Submitted filename: PNTD-D-23-00429_PLOS_article_comments2.docx [file pntd.0011493.s002.docx]

Manuscript: COVID-19 vaccination and leprosy – a UK hospital-based retrospective cohort study

Reference number: PNTD-D-23-00429

To: Linda B Adams

Academic Editor
PLOS Neglected Tropical Diseases

London, 28 June 2023

Dear Linda,

Thank you for reviewing my article. I have addressed the editorial requests and responded to the reviewers’ comments as below:

Bellow are the changes made in the manuscript:

1. Addition of 2 co-authors and their affiliations on line 2, 10, 20 and 21 and indication of corresponding author on line 12.

Line 2: “Barbara de Barros [1,2], Rachel Pierce [3], Cathryn Sprenger [4], Eugene Liat Hui Ong [4], Stephen L. Walker [1,2,3]”

Line 10: “4. Dermatology Department, St Georges University Hospitals NHS Foundation Trust, London, UK”

Line 20: “ Cathryn Sprenger: Dermatology Department, St. Georges University Hospitals, London, UK”

Line 21: “Eugene Liat Hui Ong: Consultant Dermatologist, St. Georges University Hospitals, London, UK”

Line 12: “*Corresponding author”

2. Substitution of the word and for & on line 17 in the name London School of Hygiene & Tropical Medicine.

Line 17: “ Hygiene & Tropical Medicine, London, UK”

3. In the Ethics statement on line 162.

Line 162: “Consent was not required for anonymised data.”

4. Addition of authors contributions on lines 279 to 290.

Line 285: **“Conceptualization:** Stephen L. Walker”

Line 286: **“Resources:** Barbara de Barros Rachel Pierce, Cathryn Sprenger, Eugene Liat Hui Ong and Stephen L. Walker**”**

Line 288: **“Data curation:** Barbara de Barros”

Line 289: **“Formal Analysis:** Barbara de Barros”

Line 290: **“Supervision:** Stephen L. Walker”

Line 291: **“Project administration:** Barbara de Barros and Stephen L. Walker”

Line292: **“Resources:** Barbara de Barros Rachel Pierce, Cathryn Sprenger, Eugene Liat Hui Ong and Stephen L. Walker**”**

Line 294**: “Writing – original draft:** Barbara de Barros”

Line 296: **“Writing – review and editing:** Barbara de Barros Rachel Pierce, Cathryn Sprenger, Eugene Liat Hui Ong and Stephen L. Walker**”**

Reviewers’ comments:

Methods

**Reviewer #1:** - The objectives of the study are not explicitly stated in the text. However, the study's aim appears to be to investigate whether individuals with leprosy who received the COVID-19 vaccine experienced any adverse events, including the development of leprosy or leprosy reactions within twelve weeks of vaccination. The study's findings suggest that vaccine-mediated T cell responses may be associated with the development of these adverse events in individuals with leprosy or latent M. leprae infection.

*Response: Thank you for comment. I apologise if there was any confusion regarding the objectives statement.*

*In the article line 139 we described the objectives as follow:*

*“*We wished to assess the uptake of SARS-CoV-2 vaccine amongst patients attending the Leprosy Clinic at the Hospital for Tropical Diseases (HTD), London, UK and to determine if there were any leprosy associated adverse effect.”

-yes, it is appropriate however the study's sample size is very small, the findings suggest a possible association between vaccine-mediated T cell responses and the development of leprosy or leprosy reactions in individuals with leprosy or latent M. leprae infection.
-yes, it is clearly described. 
-sample size is small and not sufficient to address the hypothesis being tested.

*Response: We acknowledge that the sample size in our study is small. As a retrospective cohort analysis, our study relied on available data from the leprosy clinic attendees during a specific period. Despite the limited sample size, we believe our findings provide valuable insights into the potential association between COVID-19 vaccination and leprosy adverse events.*

-Did not find any statistical analysis plan - simple analysis of the data was done

*Response: We apologize for not including a detailed statistical analysis plan in the manuscript. We performed a simple analysis of the data due to the retrospective nature of the study and the absence of a control group.*

-not much of a concern with ethics as it is a retrospective study and data was taken from EMR. Data collection was approved as part of a quality improvement project registered with the Infection Division of University College London Hospitals NHS Foundation Trust in accordance with institutional policy.

*Response: We appreciate your comment regarding the ethical considerations of our study. As a retrospective study using anonymised data collected as part of a quality improvement project, we ensured compliance with institutional policies and obtained appropriate approvals.*

**Reviewer #2:** Methods are clearly articulated and design is appropriate to test the hypothesis presented.
The population is clearly described and is appropriate. Sample size is not relevant as this study is analysing a retrospective cohort.
No statistical analysis is used beyond expressing data in percentages.

There was apparently no attempt to obtain individual informed consent, but if all data was extracted from clinical records and anonymised by the team responsible for clinical care of patients, in conducting this Quality Improvement exercise, then it would be reasonable not to seek individual’s consent. See lines 151-154: data extraction approved in line with institutional policy. No identifiable personal data is included and no photographs.

*Response: Thank you for your comments.*

**Reviewer #3:** The submission intends to describe leprosy reactions to occur as a potential consequence of vaccination against SARS-CoV-2. Leprosy clinic attendees were evaluated, and the study is not powered (nor is it presented as such) to support any statistical evaluation.

*Response: Thank you for your valuable feedback. We acknowledge that our study was not designed to be statistically powered or presented as such. Our objective was to assess the uptake of SARS-CoV-2 vaccine and document the occurrence of leprosy reactions among individuals who had received the vaccine. This exploratory study aimed to provide initial insights into a potential association.*

*We agree that a comprehensive statistical evaluation would be essential to establish a causal relationship between SARS-CoV-2 vaccination and leprosy reactions definitively.*
--------------------

Results

**Reviewer #1:** -no plan was presented 
-yes, results presented clearly
-yes
Response: Thank you for your feedback.

**Reviewer #2:** Result are presented clearly according to the analysis plan. There are no figures, the 2 tables are sufficiently clear.

Their findings are discussed in relation to other published reports of similar observations from endemic countries

*Response: Thank you for your review and positive feedback. We are pleased to hear that the results are presented clearly.*

**Reviewer #3:** The authors reviewed clinical records of 52 clinic attendees in 2021, reporting that 2 had leprosy or reactional episodes after receipt of the BNT162b2 vaccine (1 one week after second dose, 1 eight weeks after a single dose)

*Response: We appreciate your careful review of our study and your comments on the findings.*
--------------------

Conclusions

**Reviewer #1:** -yes, conclusions are supported by the data presented 
-The authors have clearly described the limitations of their analysis, such as the small sample size and lack of control group, which may limit the generalizability of their findings.
-Yes They have also discussed how their data can be helpful in advancing our understanding of the topic under study, specifically the potential association between COVID vaccination and leprosy adverse events.
-The authors have addressed the public health relevance of their findings by emphasizing the importance of clinicians to be aware of the potential leprosy adverse events associated with COVID vaccination. They have also highlighted the need for large-scale campaigns to ensure that individuals affected by leprosy have access to vaccination. 
Vaccination should not be delayed in individuals with leprosy.

*Response: Thank you for your insightful comments, which have helped us refine and strengthen the manuscript.*

**Reviewer #2:** The cautious conclusions are justified by the findings described. The authors explain potential relevance of findings, in relation to individuals’ and public health.

*Response: We appreciate your positive feedback on the cautiousness of our conclusions. We agree that the findings described in our study warrant a cautious approach, considering the limitations of our data and the need for further research.*

**Reviewer #3:** Conclusions are simple statements of the observations, which is appropriate given the limited data set. While attention is drawn to the 2 episodes that are clinically suggested to the related to receipt of BNT162b, and appropriate reference is made to the impact of BCG immunization on leprosy reactions, it is unclear if the observation is simply related to vaccination in general or to the COVID-19 vaccine. This reservation should be made more clear. Consideration of records and impacts of other vaccines (i.e., shingles) that might be commonly administered to this population should also be made. Further, review of historical records (pre-COVID) would provide an indication as to if 2/52 attendees an increase over typical presentation rates.

*Response: Thank you for your comments on the simplicity of our conclusions, which we agree is appropriate given the limited dataset. We acknowledge your concern regarding the clarification of whether the observation is related to vaccination in general or specifically to the COVID-19 vaccine. In the UK, there are 3 vaccines available to adults: influenza vaccine (annually and over 50 years of age) pneumococcal vaccine (over 65 years of age), and shingles vaccine (between 70 to 79 years of age). Although, the vaccines are available, not all adults receive the vaccines. For example the influenza vaccine uptake amongst GP patients in England in 2022 to 2023 was in average across all groups of 57% (*[*https://www.gov.uk/government/statistics/seasonal-influenza-vaccine-uptake-in-gp-patients-monthly-data-2022-to-2023*](https://www.gov.uk/government/statistics/seasonal-influenza-vaccine-uptake-in-gp-patients-monthly-data-2022-to-2023)*). According to a cross-sectional study by Tan et al. 67.7% of older adults in England 67.7% received the pneumococcal vaccine and 53.3% received the singles vaccine*^1^*. We have a relatively young cohort of patients, median age of 48.5 years-old, thus, they are not routinely vaccinated.*

*We appreciate your suggestion to consider the records and impacts of other commonly administered vaccines in this population. While our study focused on the potential association between leprosy reactions and the COVID-19 vaccine, we acknowledge that exploring the impact of other vaccines would be valuable and could be a direction for future research. We have added a mention of this limitation and potential avenue for future investigation in our revised manuscript.*
--------------------

**Reviewer #1:** Accept

**Reviewer #2:** Presentation does not require changing.

**Reviewer #3:** (No Response)

--------------------

Summary and General Comments

**Reviewer #1:** This study highlights a potential association between Covid vaccination and the onset of leprosy reactions and provides detailed clinical information on the cases and their outcomes. There is a comparison of findings with previous literature on vaccination and leprosy, which helps put the results in context. The study raises awareness among clinicians to consider the potential association between vaccination and leprosy reactions.
There was no control group, and the results may be subject to bias and the sample size was small, with only 14 cases identified, which limits the generalizability of the findings. 
The novelty of the study is that it reports a possible association between vaccination and leprosy reactions, specifically T1R and ENL the first study to do so. 
The study also highlights the importance of monitoring potential adverse events following vaccination and the need for further research to understand the mechanisms behind these associations.
The evidence may be of interest to the general public as it explores a potential association between leprosy adverse events and Covid vaccination. It provides important information for healthcare professionals and individuals affected by leprosy who are considering vaccination against COVID-19. However, the study has limitations, and the reported cases are rare, so it should be interpreted with caution.

**Reviewer #2:** This is an interesting report of a retrospective study of covid-19 vaccination status in leprosy affected people attending a tertiary care facility in a non-endemic country. Possibly different findings might result from a similar study in a highly endemic country with different vaccination practices
 **Reviewer #3:** The report is a straightforward presentation attempting to link COVID-19 vaccination with leprosy reactions. Wider consideration of historical trends and receipt of other vaccines would significantly elevate the study and test the hypothesis more fully.

Thank you for accepting our article for publication. We sincerely appreciate the time and effort you and the reviewers have dedicated to reviewing our work. The valuable feedback and constructive criticism provided by the reviewers will be taken in consideration in our future work.

We have carefully considered all the comments and suggestions and have made appropriate revisions to address them.

Thank you.

Best regards,

Barbara de Barros

[Barbara.de-barros@lshtm.ac.uk](mailto:Barbara.de-barros@lshtm.ac.uk)

References

1.	Tan PS, Patone M, Clift AK, et al. Factors influencing influenza, pneumococcal and shingles vaccine uptake and refusal in older adults: a population-based cross-sectional study in England. *BMJ Open*. 2023;13(3):e058705. doi:10.1136/bmjopen-2021-058705
